# Anterior Bite Opening in Adulthood

**DOI:** 10.2174/1874210601711010628

**Published:** 2017-12-13

**Authors:** Karolina Broberg, Birgitta Lindskog-Stokland, Christina Mejersjö

**Affiliations:** 1 Clinic of Orofacial Pain, University Clinics of Odontology, Public Dental Health, Region Västra Götaland, Gothenburg, Sweden; 2 Clinic of Orofacial Pain, Public Dental Health, Region Västra Götaland, Borås, Sweden; 3 University Clinics of Odontology, Public Dental Health, Region Västra Götaland, Gothenburg, Sweden

**Keywords:** Temporomandibular Dysfunction (TMD), Orofacial Pain, Bite opening, Bruxism, Myalgia, Masticatory muscles

## Abstract

**Objectives:**

To study anterior bite opening of unknown cause presenting in adulthood regarding prevalence, symptoms of Temporomandibular Dysfunction (TMD) and possible causes of the bite opening.

**Methods:**

Patients referred to two Orofacial Pain and TMD clinics with the complaint of recent anterior bite opening, presenting in adulthood and of unknown cause, were considered for the study. Patients with systemic rheumatic or neuromuscular diseases, degenerative joint disease, previous fractures of the jaws or orthodontic treatment, were excluded. The clinical examination was according to DC/TMD and extended for the occlusion. Reported symptoms, clinical signs, the occlusion and diagnoses found are presented. According to the information gained from the patient’s history, previous occlusion and appearance, and present signs of parafunction, a possible association with the bite opening was suggested.

**Results:**

Anterior bite opening was found in 1.6% of the referred patients. Symptoms of tiredness and/or orofacial pain were reported by 62%, headache by 41%, TMJ clicking by 24% and sensitive/tender teeth by 41%. Parafunction or bruxism was reported by 2/3 of the patients. A previous period in life of TMD symptoms, before the bite opening, was reported by 66%. Myalgia and headache associated with TMD were frequently diagnosed. The use of a partial dental splint, tongue pressure and pregnancy were possible causes found for the bite opening.

**Conclusion:**

Anterior bite opening can occur in adulthood without organic or systemic disease of the TMJ or masticatory muscles, and was frequently associated with muscle TMD symptoms.

## 1 INTRODUCTION

Anterior bite opening entails loss of tooth contacts and decreased overbite, causing a deterioration of the occlusion, chewing difficulties, speech difficulties, a changed appearance and a lower bite force than normal [1].

Anterior bite opening after completed growth, can occur due to degenerative changes of the temporomandibular joint (TMJ) caused by systemic rheumatic diseases or osteoarthrosis, resulting in posterior rotation of the mandible [2]. Mandibular condylar fractures [3], growth hormone disturbances, tumors in the jaws [4] and relapses after orthodontic treatment may also cause bite opening. Macroglossia can affect the jaws and result in an open bite [5], but after a surgical reduction of the tongue, the occlusion was found to improve [6]. In adults with muscular dystrophy, a neuromuscular disease, anterior bite opening commonly develops [7]. However; sometimes it is not possible to identify a cause of the adult bite opening, and the prevalence of idiopathic bite opening is not known.

The position of the teeth is influenced by forces from the lips, cheeks and tongue, and parafunctional behavior, like tongue thrusting and lip-biting, may affect the occlusion [8]. A complication of periodontitis is tooth migration and the periodontal inflammation facilitate occlusal changes from oral habits [9]. Among adults, the impact of oral forces on the occlusion is sparsely studied. Tongue thrusting has been discussed as a cause of anterior bite opening in case reports [10, 11]. Mouth breathing and juvenile swallowing patterns are other factors discussed as possible causes [8, 12]. The development of bite opening in adults with muscle dystrophy indicates the importance of muscle activity for the oral morphology [7].

The prevalence of TMD symptoms among adults requiring treatment has been estimated to 5-16% with higher values for women [13, 14]. In children and adolescents, especially in girls, an open bite has been associated with an increased risk of TMD [15], but which has not been investigated in adults. It is not known if there is an association between the bite opening and orofacial pain and dysfunction. No reports on the prevalence of anterior bite opening in adulthood were found in the literature.

The aim of this study is to investigate the prevalence and types of anterior bite opening of unknown cause in adults referred to specialist clinic, the symptoms of TMD among these patients, and to identify possible causes of anterior bite opening in adulthood.

## 2 MATERIAL AND METHODS

### 2.1 Subjects

During a two-year period, all newly referred patients to the Orofacial Pain and TMD Clinics, Public Dental Health Service in Gothenburg and Borås, Sweden, 20 years of age or older and with a subjective report of anterior bite opening presenting in adulthood were considered for a clinical observation study. An anterior bite opening was defined as a recent loss of anterior tooth contacts and reduced overbite, and confirmed by earlier patient records, available models, panoramic radiographs and cephalomteric radiographs, photos of the occlusion at the patient´s general practitioner, earlier photos of the patient’s facial appearance, and facets on incisors/canines that could not be reached with the current occlusion.

Patients with TMJ arthrosis, systemic rheumatic diseases, neuromuscular diseases, previous fractures of the jaws and recent prosthetic or orthodontic treatment, were excluded in order to get a group of patients with no known cause of the bite opening. During a two-year period, 1889 new consecutive patients (≥ 20 years old) were examined at the two clinics, and of those, 34 patients matched the inclusion criteria. Four of these patients were excluded after the TMJ radiography due to degenerative joint changes, not suspected from the clinical examination. Informed consent to participate was obtained from each patient, one patient rejected inclusion and 29 patients were included in the study. The reason for referral was occlusal problems in 52%, and occlusal problems and pain in 48%.

### 2.2 Examinations

The patients first completed a standardized questionnaire in accordance with the Diagnostic Criteria for Temporomandibular Disorders (DC/TMD) [16] and regarding their general health. In order to catch information about circumstances able to give a bite opening, the patient’s history was supplemented by an interview regarding the bite opening, any previous orthodontic or prosthodontic treatment and a medical history. The patients classified the degree of their current reported symptoms (Si) on a verbal five-point scale (1 = no/minimal, 2 = light, 3 = moderate, 4 = fairly severe, 5 = severe symptoms [17]). Unfortunately, there was a delay of 6-12 months from the referral until the examinations due to a large number of referrals.

A clinical examinations according to the DC/TMD [16] was performed and with an extension for the occlusion [18], the same examiner for all the patients. The examiner was used to and trained with the DC/TMD examination, and the diagnosis of TMD was stated according the DC/TMD. Clinical signs of parafunctional behavior, and the appearance and location of mucosal impressions from the teeth on the cheeks, lips and tongue, were noted. Impressions on the upper surface of the tongue from the occluding teeth was regarded a sign of tongue pressure in between the dental aches. According to the information gained from the patient’s history, previous occlusion and appearance, and present signs of parafunction, a possible association with the bite opening was suggested for each patient.

Occlusal contacts were recorded in the maximum intercuspal position (ICP), tooth by tooth of the maxilla by using a plastic colored foil (Trollfoil, Troll Dental, 8μm) attached to a Miller articulating forceps tweezers. The opening width, defined as the number of teeth in a row without an occluding contact, was measured for each patient. The maxillary dental arch width was measured according to Kiliaridis *et al.*, 2003 [19]. For patients using a splint, the present and former splints were examined with regard to type, occlusal coverage and contacts.

Radiographic examination of the TMJ was performed with cone beam computed tomography (CBCT) according to the routine at the clinics for investigating occlusal changes. Only patients with normal TMJs were included in the study to ensure a group of patients without a known possible cause of the anterior bite opening. Dentofacial morphology was assessed on digital lateral radiographs taken in central occlusion. The radiographs were traced using a cephalometric software (FACAD, Illexis AB, Linköping, Sweden). Measurement points and reference lines defined by Bjork [20] are shown in Fig. (**1**).

### 2.3 Ethical Aspects

The questionnaire and the examinations followed the routines at the clinics´ for investigation of patient´s occlusal changes. Informed consent to participate in the study was obtained from each patient and the guidelines of the Helsinki Declaration have been followed. The study was discussed with the Ethical Committee at the University of Gothenburg, and according to their written policy for this type of studies, no more ethical approval was required.

### 2.4 Statistical Analyses

The statistical analyses were performed by using the IBM SPSS Statistics 22 software (SPSS Inc., Chicago, Il). For the analysis of differences between the groups, Studentʼs t-test and the Mann-Whitney U-test were used, with the p-value < .05 for statistical significance. As there are only few patients with anterior bite opening, and no previous study of adult bite opening was found, there was no power analysis performed and the study could be regarded as a pilot study to get some information of these patients.

## 3 RESULTS

The prevalence among referred patients of recent anterior bite opening in adulthood was 1.6%. The patients (23 women and 6 men) were aged 21-71 years (= 36.2, SD 12.1). The degree of bite opening varied, and the over bite ranged from +1.5 to -4 mm. For the whole group, the mean number of occluding pairs of teeth in ICP was 3.7 (SD ±1.5), the mean opening width was 11 teeth in a row without occlusal contact (SD ±2.2), and the mean dental arch width was 32.8 mm (SD ± 2.98). The patients’ occlusion is presented in Table **1**.

**Fig. (2):**
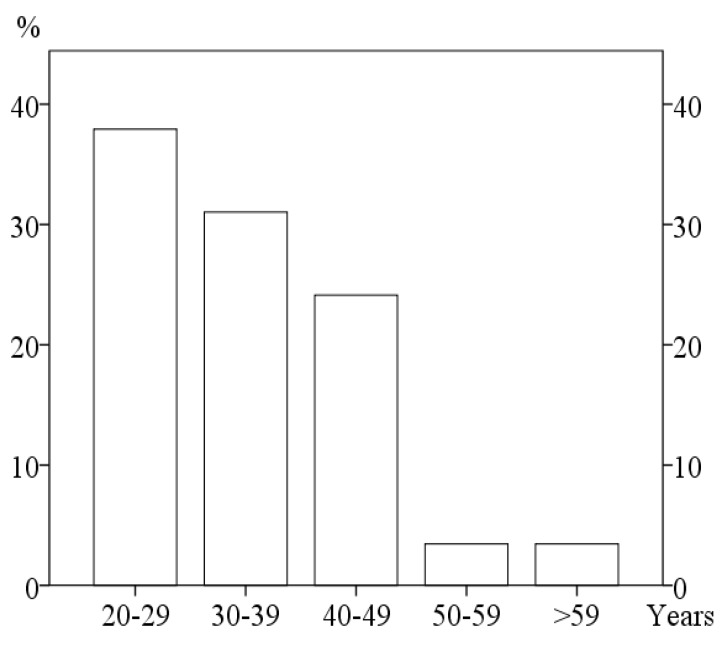
The patients age at start of the anterior bite opening.

The bite opening had developed after the age of 30 in 62% of the patients (Fig. **2**) and had started less than three years before the examination in 69%. In most patients, the bite opening had developed slowly.

A cephalometric analysis was performed in 21 patients (Table **2**). Some structures were however, not visible in all radiographs which explain the smaller number of patients (n) for some variables. The cephalometric values were considered to be within a normal range except the maxillary plane (NSL/NL angle) which on average was 5.2 degrees (SD= 2.2), implying that the maxilla was more anterior rotated compared to a normal group [20].

### 3.1 Reported Symptoms

Symptoms of pain and dysfunction of the jaws and head once a week or more often, were reported by 62% for tiredness and/or orofacial pain, 41% for headache, 24% for clicking, 3% for difficulty opening wide, and 41% for sensitive/tender teeth. Parafunction or bruxism was reported by 2/3 of the patients and only four denied such activity. According to the subjective index, 62% of the patients evaluated their symptoms as moderate to severe. A previous period in life of TMD symptoms or treatment of bruxism before the bite opening, was reported by 66%.

### 3.2 Clinical Signs and Diagnoses

Muscle tenderness on palpation was the most common clinical finding, with 79% of the patients fulfilling the criteria for myalgia. Headache associated with TMD was diagnosed in 41% and arthralgia in two patients (7%). TMJ disc displacement was diagnosed in nine patients, but only one patient had current symptoms other than the sound. The mean maximum mouth opening capacity was 53 mm (37-68), only one was regarded impaired. All patients but one had some type of dental impression on the tongue, lips and/or buccal mucosa.

### 3.3 Possible Associations with the Bite Opening

Seven of the patients (24%) used, or had been using, a splint not covering the last upper molars. Four patients (14%) reported that the bite opening had started during pregnancy, and associated with body edema and breathing difficulties for two of them. The other 18 patients (62%) all had tongue impressions of the type assumed to indicate a habit of keeping/pressing the tongue between the dental arches.

A significant difference (*p* = 0.008) was noticed for the occlusal opening width between the group of possible tongue pressure (mean 10.3 teeth in a row, SD ±2.1) and the group of a use of a partial splint (mean 12.6 teeth, SD ± 1.2). Six patients (34%) in the group of possible tongue pressure had a cross bite, while none of the patients with the other suggested causes. There was no significant difference between the different possible causes and the symptoms of TMD, dental arch width, cephalometric values, age or gender.

## 4 DISCUSSION

A prevalence of 1.6% of anterior bite opening of unknown cause was found among the adult patients referred to the two Orofacial pain and TMD Clinics. There are probably more hidden cases of anterior bite opening in adulthood in the population, and those without pain may not be discovered. The confirmation of a recent bite opening was based on a combination of factors indicating the change of occlusion, besides the information from the patient about a loss of tooth contacts and the ability to bite off at the front teeth, which ensures that the study comprises patients with a bite opening presenting after the growth had been completed.

In order to ensure a group of patients without a known cause of the bite opening, patients with a rheumatic or neuromuscular disease, and TMJ osteoarthrosis were not included as the bite opening could be a result of the disease. However, degenerative changes or remodeling of the TMJ could also be a consequence of an altered loading of the TMJs and loss of occlusal contacts due to a bite opening [21].

The development of an anterior open bite could be explained by over-eruption of the last molars as due to a splint not covering those teeth. Another possible cause is a tongue pressure between the mandible and maxillary arches when the tongue covers most of the occlusal surfaces but not reaches the posterior molars, and giving the last molars a possibility to over erupt [22]. A changed tongue behavior could originate from protecting a tender tooth, or maybe from a dentist’s remark about attrition and the patient´s desire to protect the teeth from non-functional biting. The fact that the maxilla was more anteriorly rotated in most of the cases might even implicate that the tongue pressure could initiate a skeletal change as part cause to the anterior bite opening. Cross-bites were more often found in the group of suspected tongue pressure, and the absence of a tongue position in the palate could influence the morphology of the maxilla and the development of a cross bite.

Myalgia and headache associated with TMD were frequently diagnosed, and a majority of the patients had previous periods in life of TMD symptoms. This indicates an association between muscle tension and the development of a bite opening. Facets on incisors and canines were clinical signs that confirmed previous occlusal contacts, but this could also indicate a previous bruxing behavior. A non-functional muscle activity of bruxism had changed towards a more tongue- and lip-pressing behavior causing the bite opening.

A bite opening leads to an open bite, which is a malocclusion that may lead to neuromuscular changes [23]. It is not possible to say whether the TMD symptoms among the patients of the study, are associated with the impaired occlusion following the bite opening, or if both the TMD symptoms and the occlusal changes are results of an increased or altered parafunctional activity. Sensitive or tender teeth were reported by 41% of the patients, which could relate to the distribution of the occluding forces across fewer teeth than normally. However, bruxism and parafunctional activity could also give both myalgia and hypersensitive teeth.

The study shows that the occlusion can easily be altered in adults by a splint with partial extension, but factors influencing the occlusion in adulthood are not fully known. However, a patient should not be left with a split for a longer period of time without check-ups.

Tooth migration is associated by gingival inflammation [24], and during pregnancy there was a significant increase of the gingival inflammation in healthy women [25]. Three patients of the study noticed the start of their bite opening during pregnancy and another patient (nr 8) had jaw problems during her pregnancy but could not remember if her bite opening stated during or after the pregnancy. Two of these women complained of body edema and breathing difficulties during pregnancy.

## CONCLUSION

Anterior bite opening can occur in adulthood without organic or systemic disease of the TMJs or masticatory muscles, and was frequently associated with muscle symptoms. A majority of the patients had previous periods of TMD symptoms before the bite opening started. A partial splint and oral parafunctional activity is a possible cause of anterior bite opening after complete growth.

## ETHICS APPROVAL AND CONSENT TO PARTICIPATE

The study was discussed with the Ethical Committee at the University of Gothenburg, and according to their written policy for this type of studies, no more ethical approval was required.

## HUMAN AND ANIMAL RIGHTS

The reported experiments in accordance with the ethical standards of the committee responsible for human experimentation (institutional and national), and with the Helsinki Declaration of 1975, as revised in 2008 (http://www.wma.net/en/20activities/10ethics/10helsinki/).

## CONSENT FOR PUBLICATION

Not applicable.

## Figures and Tables

**Fig. (1):**
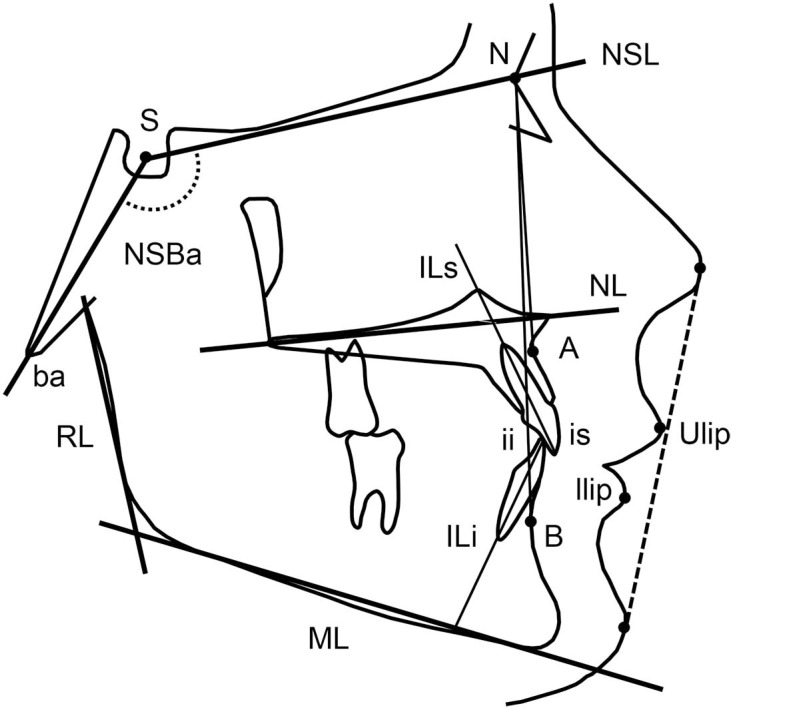
Cephalometric landmarks and lines.

**Table 1:** Clinical findings in 29 adult patients with anterior bite opening (F = female, M = male, ICP = intercuspal position, transversal relation = neutral if nothing is said,

Pat. No	Age (Years)	Overbite (mm)	Angle Class	Transvers.	Maxillary Teeth Without Occluding Contact in ICP (○)																Maxillary Dental Arch Width (mm)	Possible Cause
				Relation	Maxillary Teeth with Occluding Contact in ICP (●)																	of Bite
					18	17	16	15	14	13	12	11	21	22	23	24	25	26	27	28		Opening
1	50	-2.0	II		●	●	●		○	○	○	○	○	○	○	○		●	●		35.4	Tongue
2	42	0	I		●	●	○	○	○	○	○	○	○	○	○	○	○	○	●	●	34.6	Splint
3	49	0	I			●	○	○	○	○	○	○	○	○	○	○	○	○	●		-	Splint
4	21	0.5	I		○	●	●	○	○	○	○	○	○	○	○	○	○	●	●	●	32.2	Tongue
5	44	-1.0	I	Cross bite, unilat.	●	●	○	○	○	○	○	○	○	○	○	○	○	○	●	○	33.3	Tongue
6	27	0	I		●	○	○	○		○	○	○	○	○	○		○	○	○	●	29.4	Splint
7	32	1.0	I		●	●	●	○	○	○	○	○	○	○	○	○	○	●	●	○	31.8	Tongue
8*	44	-0.5	I		●	●	●	●	●	○		○	○		○	○	○	●	●	○	-	Pregnancy
9	25	-1.0	I		●	○	○	○	○	○	○	○	○	○	○	○	○	○	○	●	35.8	Splint
10	48	-2.5	I	Cross bite, unilat.		●	●	●	○	○	○	○	○	○	○	○	○	●	●		31.7	Tongue
11*	33	-1.0	I		●	○	○	○	○	○	○	○	○	○	○	○	○	○	○	●	-	Pregnancy
12	58	1.0	I		○	●	○		○	○	○	○	○	○	○	○	●	●	●		29.6	Tongue
13	36	-1.0	I		●	●	○	○	○	○	○	○	○	○	○	○	○	○	●	●	33.5	Splint
14	37	-3.0	II	Cross bite, unilat.	○	●	●	○		○	○	○	○	○	○		○	●	●	○	26.5	Tongue
15	35	0.5	I	Cross bite, unilat.	●	○	○	○	○	○	○	○	○	○	○	○	○	○	●	●	34.0	Tongue
16	41	-1.0	I		●	○	○	○	○	○	○	○	○	○	○	○	○	○	○	●	36.2	Splint
17	24	-1.0	I		●	●	○	○	○	○	○	○	○	○	○	○	●	●	●	●	28.7	Tongue
18	43	1.0	I		●	●	○	○	○	○	○	○	○	○	○	○	○	○	○	●	31.6	Tongue
19	25	-1.0	I		○	●	●	○	○	○	○	○	○	○	○	○	○	○	○	○	37.0	Tongue
20	71	-3.0	I	Cross bite, bilat.		●	●	○	○	○	○	○	○	○	○	○	○	●	●		36.8	Tongue
21	25	-0.5	II			●	○	○		○	○	○	○	○	○	○	○	○	●		31.7	Tongue
22	23	-0.5	I			●	○	○	○	○	○	○	○	○	○	○	○	●	●		34.9	Splint
23	37	-4.0	II		●	●	○	○	○	○	○	○	○	○	○	○	○	○	○	●	30.1	Tongue
24	24	0	I	Cross bite, unilat.	○	○	○	○	○	●	○	○	○	○	○	○	○	○	●	●	34.6	Tongue
25	24	1.5	I			●	●	●	○	○	○	○	○	○	●	●	●	○	○		34.7	Tongue
26*	27	-1.0	I		●	●	●	○	○	○	○	○	○	○	○	○	○	●	●	●	34.0	Breathing diff.
27*	34	-1.0	I		●	○	○	○		○	○	○	○	○	○		○	○	○	●	30.0	Breathing diff.
28	47	-1.0	I		●	●	○	○	○	○	○	○	○	○	○	○	○	○	●	●	37.3	Tongue
29	23	-3.0	I		●	●	○	○		○	○	○	○	○	○		○	○	○	●	27.2	Tongue

* pregnancy at start of the bite opening)

**Table 2:** Cephalometric analysis.

Variable	n	Norm	Mean	SD	Max	Min
SNA	21	82±3	83,0	3,9	89,5	75,5
SNB	19	80±3	78,9	4,4	86,8	70,6
ANB	19	3±2	3,6	2,5	10,2	-0,2
NSBa	21	130±5	129,0	5,6	141,4	119,3
NSL/NL	21	9±3	5,8	2,2	9,6	1,4
NSL/ML	17	33±4	32,2	6,2	42,3	20,0
NL/ML	17	24±4	26,3	5,7	36,0	14,3
ML/RL	17	124±6	125,4	7,5	142,8	109,4
ILs/NL	21	110±5	107,0	7,5	118,0	94,7
ILi/ML	17	95±5	94,2	7,1	104,4	80,7
Low fac %	17	71%-89%	74,6	5,4	83,4	62,8
Interincisal	19	131±10	132,2	10,9	159,5	114,4
ILs/NA	21	22±8	18,2	7,3	32,5	3,7
ILi/NB	19	25±8	25,6	6,6	33,9	7,5
Upper lip - E-line	16	-4±2	-6,3	2,0	-2,0	-10,0
Lower lip - E-line	16	-2±2	-4,4	2,2	-0,9	-8,7
